# A Kalman Filter for SINS Self-Alignment Based on Vector Observation

**DOI:** 10.3390/s17020264

**Published:** 2017-01-29

**Authors:** Xiang Xu, Xiaosu Xu, Tao Zhang, Yao Li, Jinwu Tong

**Affiliations:** 1 Key Laboratory of Micro-Inertial Instrument and Advanced Navigation Technology, Nanjing 210096, China; xuxiang@seu.edu.cn (X.X.); zhangtao22@seu.edu.cn (T.Z.); liyao@seu.edu.cn (Y.L.); tongjinwu@seu.edu.cn (J.T.); 2 Ministry of Education, School of Instrument Science and Engineering, Southeast University, Nanjing 210096, China

**Keywords:** strapdown inertial navigation system, self-alignment, Kalman filter, parameter recognition and reconstruction

## Abstract

In this paper, a self-alignment method for strapdown inertial navigation systems based on the ***q***-method is studied. In addition, an improved method based on integrating gravitational apparent motion to form apparent velocity is designed, which can reduce the random noises of the observation vectors. For further analysis, a novel self-alignment method using a Kalman filter based on adaptive filter technology is proposed, which transforms the self-alignment procedure into an attitude estimation using the observation vectors. In the proposed method, a linear psuedo-measurement equation is adopted by employing the transfer method between the quaternion and the observation vectors. Analysis and simulation indicate that the accuracy of the self-alignment is improved. Meanwhile, to improve the convergence rate of the proposed method, a new method based on parameter recognition and a reconstruction algorithm for apparent gravitation is devised, which can reduce the influence of the random noises of the observation vectors. Simulations and turntable tests are carried out, and the results indicate that the proposed method can acquire sound alignment results with lower standard variances, and can obtain higher alignment accuracy and a faster convergence rate.

## 1. Introduction

Initial alignment is a crucial procedure of strapdown inertial navigation systems (SINS), and high precision of the initial alignment for SINS is needed to keep the stability of SINS [1,2,3]. The traditional initial alignment can be divided into two major categories: one is the transfer alignment, which needs external information from other navigation devices; the other one is self-alignment, which can accomplish the initial alignment by using the gravitational attraction and the self-rotation of the Earth [4]. Within the frame of the former category, it may be achieved quite simply by the direct copying of data from the external navigation system, and more precisely with methods of an inertial measurement matching process, but this requires other complex systems and may ignore the self-contained advantages of SINS. Thus, the self-alignment method, which is the second category, is the hot topic of SINS, and many researchers are devoted to improving the performance of the self-alignment.

Typically, the traditional self-alignment method can be accomplished by two stages, i.e., the coarse alignment stage and the fine alignment stage. Within the fine alignment stage, the initial attitude of SINS can be acquired more accurately and, furthermore, other information, such as sensor biases and initial velocity, can be estimated simultaneously [5,6,7]. However, the fine alignment stage can be proceeded by the linear Kalman filter only when the error state model is confined to a small misalignment angle, which can be provided by the coarse stage. With the coarse alignment, the rough attitude of the vehicle can be acquired. In the coarse alignment stage, it is assumed that the position of the vehicle is well known. Then, the Earth’s rotation and gravity can be computed accurately, and the estimation of the initial attitude can be acquired by comparing the computed rotation rates and gravity to the sensed and rated acceleration. Due to the significance of the coarse alignment, the performance of fine alignment can be improved by excellent coarse alignment. Thus, if there is a method which can be better implemented than the traditional coarse alignment in terms of convergence rate, alignment accuracy, and stability of the alignment results, it will be a novel self-alignment which can be applied in some emergency situations, since the fine alignment always needs 20 to 30 min, sometimes more. This is what this paper focuses on.

Many methods have been devised to improve the performance of the self-alignment method, and the common method was dual-vector attitude determination based on the gravitational apparent motion in the inertial frame [8,9,10,11,12,13,14]. As is well known, the swaying motion of the Earth is composed of two distinct elements: first, the true inertial swaying caused by the motion of the body frame relative to the inertial frame; and second, the apparent angular rate caused by the self-rotation of the Earth. Based on the properties of the swaying motion, Lian and Yan [8,9] proposed a method based on the dual-vector attitude determination, and because the acceleration measured by the inertial measurement unit (IMU) axes contains random noises, which contaminate the observation vectors, the alignment time was prolonged and the alignment accuracy was decreased. To address these issues, a reconstruction algorithm for the observation vectors was proposed in [10,11,12]. By adopting the reconstructed observation vectors, the random noises were effectively suppressed. Therefore, the performance of the self-alignment was improved. In [13], a similar self-alignment based on a fixed integral sliding method was investigated. Lu et al. [14] extended the method in [13] to the optimal parameter self-alignment. However, the above dual-vector attitude determination method is not a recursive algorithm, and it is a single-point attitude determination algorithm; that is, it utilizes the observation vectors obtained at two time points and uses them, and only them, to determine the attitude at one time point. With this method, the information contained in the past measurements is lost. In order to take full advantage of all of the observation vectors, a quaternion self-alignment method based on the ***q***-method was developed in [15], which utilized the filter quaternion estimation (QUEST) method to process the observation vectors recursively [16]. Meanwhile, the optimal initial attitude could be calculated by the attitude-quaternion which was extracted by the Newton iteration algorithm from the constructed ***K***-matrix [17]. Compared with the dual-vector attitude determination method, the convergence rate and stability of the self-alignment based on the ***q***-method was improved [18,19,20]. However, the quaternion self-alignment also contained the random noises in the observation vectors, and these defects, which are not beneficial to the self-alignment, have contaminated the observation vectors. In order to denoise the observation vectors and ensure high computational efficiency, a new quaternion self-alignment method using apparent velocity has been designed in this paper; it was inspired by [21]. Since the integration process averages the measurements over a period of time, the effect of the random noises is reduced and, hence, a stable attitude estimate is obtained. In this paper, this improved scheme is considered as a comparison method for the more advanced self-alignment method.

According to the minimal-variance theory [22], none of the aforementioned self-alignment methods are optimal. Hinging on the previous results about the quaternion Kalman filter with pseudo-measurements, a Kalman filter is developed, along with a computationally simpler adaptive filtering theory [23,24]. Furthermore, the Kalman filtering approach yields, by design, sequential quaternion estimations that are minimum-variance and allows for the estimation of parameters other than attitude in a straightforward manner [22]. In this work, we develop a novel quaternion self-alignment method. Firstly, the present work introduces a linearized quaternion observation model, and the measurement noise is state-dependent. Nevertheless, the state-dependence of the measurement model induces modeling errors, which will cause filtering divergence. Then, a Kalman filter is designed to deal with the special measurement noises, which is based on adaptive filtering technology. A nice feature of the quaternion self-alignment is that the estimated quaternion is constant, and the computed observation vector has slow-varying characteristics. Therefore, a parameter recognition and observation vector reconstruction algorithm is designed to improve the convergence rate in this work. According to the brute-force normalization of the estimated quaternion, the stability of the filter is improved. Lastly, the performance of this novel self-alignment is firstly checked under the nominal simulated conditions of noise and initial errors, and then the on-line turntable tests are designed to verify the stability and accuracy of the proposed method.

In the following section we will give the principles of the quaternion self-alignment method based on the ***q***-method, and an improved method based on apparent velocity is designed. In addition, the merits and demerits are also analyzed with simulations. Next, we will describe the novel quaternion self-alignment based on the Kalman filter in detail, and a simulation is designed to evaluate the algorithm. In Section 4, an improved algorithm based on the novel quaternion self-alignment is developed, and the merits of the methods are analyzed with simulations. Turntable tests are carried out to verify the effectiveness of this novel algorithm in Section 5. Finally, the major contributions of this paper are summarized in Section 6.

## 2. General Quaternion Self-Alignment

The definitions of the coordinate frames used in this paper are described in Appendix A. It is well-known that the acceleration measured by inertial measurement unit (IMU) axes (*b*-frame) is composed of the true inertial acceleration of the vehicle caused by the motion of the *b*-frame relative to the *i*-frame and the apparent acceleration caused by the gravitational attraction of the Earth. The former element can be compensated by external sensors, such as the Doppler velocity log (DVL) and the global positioning system (GPS), and the apparent acceleration in the *b*0-frame, which is named the observation vector, can be calculated by the acceleration measurements and the gyroscope measurements. That is how the true inertial acceleration is compensated. Taking advantage of the known position, the true gravity in the *n*0-frame, which is named the reference vector, can be obtained accurately. In this section, general quaternion self-alignment using the ***q***-method is investigated, and the filter QUEST technology is designed to realize the recursive algorithm. In order to improve the stability of general quaternion self-alignment, an improved method based on apparent velocity technology is designed.

### 2.1. Mechanism of General Quaternion Self-Alignment

With the rules of quaternion multiplication, the attitude quaternion $q_{b}^{n}\left(t\right)$ from the *b*-frame to the *n*-frame can be expressed as:

(1) $$q_{b}^{n}\left(t\right)=q_{n0}^{n}\left(t\right)⊗q_{b0}^{n0}⊗q_{b}^{b0}\left(t\right)$$

where $q_{b0}^{n0}\in {\mathbb{R}}^{4\times 1}$ is the unknown initial attitude quaternion, and the symbol ⊗ used hereafter represents the quaternion product. According to the quaternion kinematic equations, the time-varying quaternion can be updated as:

(2) $$\{\begin{array}{c} {\dot{q}}_{n}^{n0}\left(t\right)=\frac{1}{2}q_{n}^{n0}\left(t\right)⊗\omega_{in}^{n}\\ {\dot{q}}_{b}^{b0}\left(t\right)=\frac{1}{2}q_{b}^{b0}\left(t\right)⊗\omega_{ib}^{b} \end{array}$$

where $q_{n}^{n0}\left(0\right)=q_{b}^{b0}\left(0\right)={\left[1\;0\;0\;0\right]}^{T}$, $\omega_{ib}^{b}\in {\mathbb{R}}^{3\times 1}$ is the rotational rates measured in the *b*-frame with respect to the *i*-frame, and it can be acquired from the gyroscope directly; $\omega_{in}^{n}\in {\mathbb{R}}^{3\times 1}$ indicates the rotational rates measured in the *n*-frame, and its theoretical expression is defined as:

(3) $$\omega_{in}^{n}=\omega_{ie}^{n}+\omega_{en}^{n}$$

where $\omega_{ie}^{n}\in {\mathbb{R}}^{3\times 1}$ is the Earth’s rotation rate with respect to the inertial frame, and $\omega_{en}^{n}\in {\mathbb{R}}^{3\times 1}$ denotes the angular rate of the navigation frame with respect to the Earth’s frame. All of the aforementioned parameters can be calculated by the outputs of the GPS. All of the quantities above are functions of time, and, if not stated, their time dependences are omitted for brevity in the following description.

It is noted that the attitude quaternions $q_{n}^{n0}\left(t\right)$ and $q_{b}^{b0}\left(t\right)$ can be calculated by Equation (2). If the initial attitude-quaternion $q_{b0}^{n0}$ is calculated, self-alignment can be accomplished. 

The apparent velocity update equation in the *n*-frame is known as:

(4) $${\dot{v}}^{n}=f^{n}-\left(2\omega_{ie}^{n}+\omega_{en}^{n}\right)\times v^{n}+g^{n}$$

where $f^{n}={\left[\begin{array}{ccc} f_{E} & f_{N} & f_{U} \end{array}\right]}^{T}$ denotes the specific force vector, which is described by the *n*-frame. Further, $v^{n}={\left[\begin{array}{ccc} v_{E} & v_{N} & v_{U} \end{array}\right]}^{T}$. $g^{n}={\left[\begin{array}{ccc} 0 & 0 & g \end{array}\right]}^{T}$ is the local gravity.

Because quaternion multiplication can be used in place of matrix multiplication to transform a three-component vector from the *b*-frame to the *n*-frame, then:

(5) $$f^{n}=q_{b}^{n}\left(t\right)⊗f^{b}⊗{\left(q_{b}^{n}\left(t\right)\right)}^{*}$$

where * denotes the conjugate operation of a quaternion, and $f^{b}$ can be measured by the accelerometer.

Substituting Equations (1) and (5) into Equation (4) yields:

(6) $$q_{b}^{b0}⊗f^{b}⊗{\left(q_{b}^{b0}\right)}^{*}=q_{n0}^{b0}⊗q_{n}^{n0}⊗\left({\dot{v}}^{n}+\left(2\omega_{ie}^{n}+\omega_{en}^{n}\right)\times v^{n}-g^{n}\right)⊗{\left(q_{n}^{n0}\right)}^{*}⊗{\left(q_{n0}^{b0}\right)}^{*}$$

Defining the reference vector and observation vector as:

(7) $$\{\begin{array}{c} r=q_{n}^{n0}⊗\left({\dot{v}}^{n}+\left(2\omega_{ie}^{n}+\omega_{en}^{n}\right)\times v^{n}-g^{n}\right)⊗{\left(q_{n}^{n0}\right)}^{*}\\ o=q_{b}^{b0}⊗f^{b}⊗{\left(q_{b}^{b0}\right)}^{*} \end{array}$$

Equation (6) can be rewritten as the observation vectors–based measurement model for $q_{n0}^{b0}$ as:

(8) $$o=C\left(q_{n0}^{b0}\right)r$$

**Figure 1:**
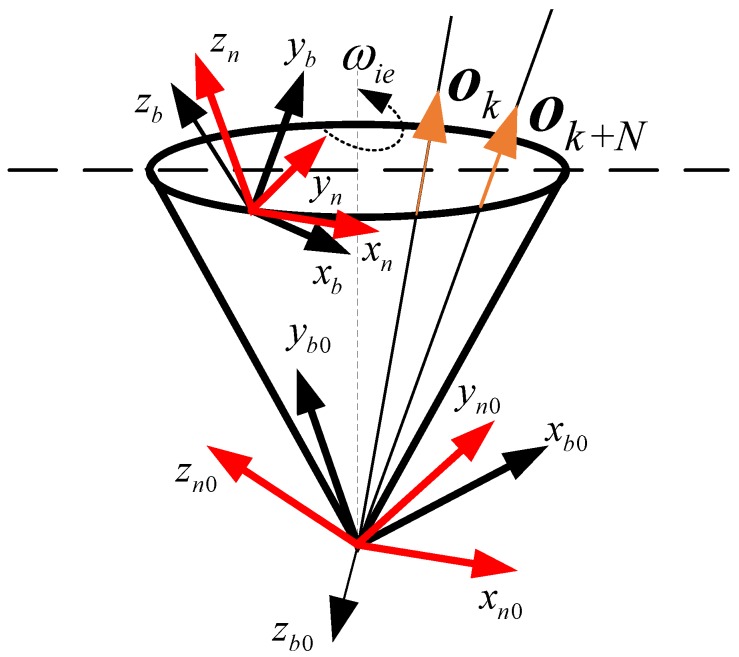
The general quaternion self-alignment mechanism based on observation vectors.

Figure 1 shows the quaternion self-alignment mechanism based on the observation vectors; the frame in red represents the *n*-frame and the frame in black represents the *b*-frame. In the self-alignment process, the observation vectors o and reference vectors r can be calculated over a period of sampling time, and the quaternion $q_{b0}^{n0}$ can be computed in real time. With the known time-varying quaternions $q_{n}^{n0}\left(t\right)$ and $q_{b}^{b0}\left(t\right)$, which can be obtained by Equation (2), the attitude quaternion $q_{b}^{n}\left(t\right)$ of the *b*-frame with respect to the *n*-frame can be calculated by Equation (1). In the following subsection, the traditional method for calculating quaternion $q_{b0}^{n0}$ is introduced.

### 2.2. Attitude Determination Based on the q-Method

According to the ***q***-method, the observation vectors and reference vectors must be normalized, respectively, to keep the normalization characteristic of the constructed ***K***-matrix [25]; it has:

(9) $$\{\begin{array}{c} \alpha_{k}=\frac{r_{k}}{∥r_{k}∥}\\ \beta_{k}=\frac{o_{k}}{∥o_{k}∥} \end{array}$$

where $\alpha_{k}\in {\mathbb{R}}^{3\times 1}$ and $\beta_{k}\in {\mathbb{R}}^{3\times 1}$ are the normalized reference vectors and the normalized observation vectors, respectively. The subscript k is the discretization scale coefficient.

The normalized ***K***-matrix is defined as follows:

(10) $$K_{k}=\left[\begin{array}{cc} \sigma_{k} & z_{k}^{T}\\ z_{k} & S_{k}-\sigma_{k}I_{3} \end{array}\right]$$

where:

(11) $$\{\begin{array}{cc} \Upsilon_{k}=\frac{k-1}{k}\Upsilon_{k-1}+\frac{1}{k}\beta_{k}\alpha_{k}^{T} & \sigma_{k}=tr\left(\Upsilon_{k}\right)\\ \left[z_{k}\times \right]=\Upsilon_{k}^{T}-\Upsilon_{k} & S_{k}=\Upsilon_{k}^{T}+\Upsilon_{k} \end{array}$$

where $\Upsilon_{0}\in {\mathbb{R}}^{3\times 3}$ is an arbitrary vector, tr(·) denotes the trace operation, [⋅×] denotes the cross-product matrix. It was shown that $q_{b0}^{n0}$ of unity length and $K_{k}$ satisfies the equation [26]:

(12) $$K_{k}{q_{b0}^{n0}}_{k}=\lambda_{max}{q_{b0}^{n0}}_{k}$$

where the subscript k of ${q_{b0}^{n0}}_{k}$ denotes the $k^{th}$-step calculation. It is well-known that $\lambda_{max}$ is the maximum eigenvalue of $K_{k}$ and ${q_{b0}^{n0}}_{k}$ is the eigenvector which corresponds to $\lambda_{max}$.

### 2.3. An Improved Algorithm for General Quaternion Self-Alignment

In Equation (11), it can be found that there is more effective observation information in the K-matrix during the alignment process. Due to the sensitivity of the filter QUEST to random noise, the stability of the results must be poor if the accelerometer measurements are used to construct the observation vector straightforwardly. In order to improve the stability of the alignment results, an improved algorithm based on apparent velocity is designed.

According to Equation (8):

(13) $$\int_{0}^{t}o\left(\tau \right)d\tau =C\left(q_{n0}^{b0}\right)\int_{0}^{t}r\left(\tau \right)d\tau$$

The discrete form of the continuous integration is given by:

(14) $$\{\begin{array}{c} \int_{0}^{t}o\left(\tau \right)d\tau =Ο\left(0\right)+\overset{N}{\underset{k=1}{\sum }}o_{k}\Delta t\\ \int_{0}^{t}r\left(\tau \right)d\tau =R\left(0\right)+\overset{N}{\underset{k=1}{\sum }}r_{k}\Delta t \end{array}$$

where $Ο\left(0\right)\in {\mathbb{R}}^{3\times 1}$ and $R\left(0\right)\in {\mathbb{R}}^{3\times 1}$ are both set to zero at start-up, because the extracted attitude quaternion between two frames is only related to the directions of the two vectors, and it has no relation to their location when the attitude determination algorithm is used. Then, the new observation vectors Ο(k) and reference vectors R(k) can be acquired by Equation (14), and the attitude quaternion $q_{b0}^{n0}\left(k\right)$ at start-up can be recalculated by the filter QUEST method.

### 2.4. Simulation Test

In this subsection, a simulation test is designed to validate the performance of the general quaternion self-alignment. The whole self-alignment process lasted for 600 s, and the geographic latitude and longitude of the vehicle were L=32°N and θ=118°E. During the simulation test, the vehicle (taking the ship as an example) was assumed to be static but with swinging motions. The swaying rule is Asin(2πft+φ)+θ, where A and f are the amplitude and frequency of the swaying, while φ and θ denote the initial phase and swaying center, respectively. The swaying parameters of this simulation are defined in Table 1:

With these ideal motions in Table 1, the truth measurement of the gyroscope and accelerometer can be simulated by the back-stepping of the SINS solution. When the errors in Table 2 are added into those ideal measurement data, the real inertial sensor outputs can be generated, and the sampling rate of the inertial sensors was 100 Hz in this test. At the same time, those ideal motions can be used as a reference to evaluate the accuracy of the alignment, and the difference between those ideal motions and the alignment results are defined as alignment errors in this paper.

**Figure 2:**
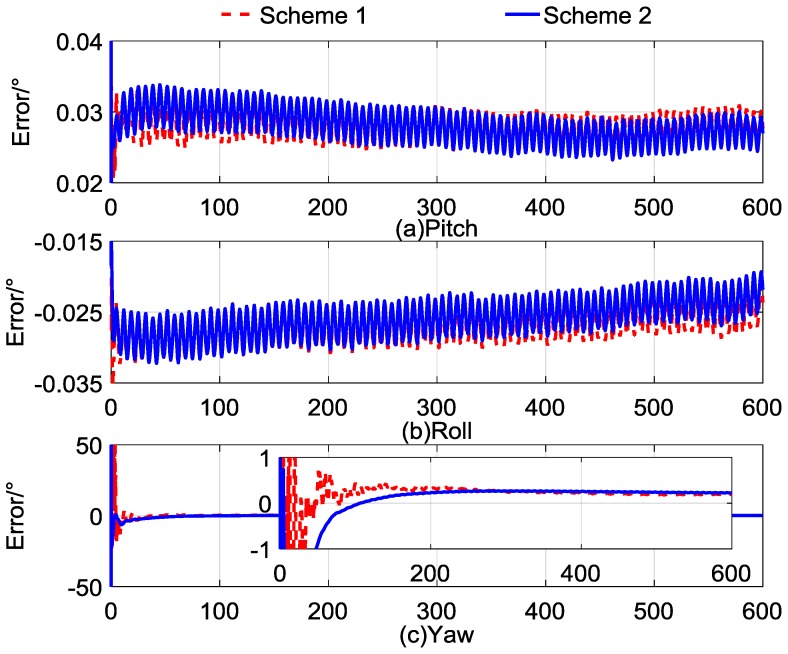
Curves of self-alignment errors.

For clarity, we define the general quaternion self-alignment method based on Equation (7) as Scheme 1, and we define Equation (14) as Scheme 2. The alignment results are recorded continuously, and the errors of the alignment results are calculated and saved as text as well. The alignment errors are shown in Figure 2, and the statistics of the alignment errors are listed in Table 3. The errors of pitch, roll, and yaw are denoted as Figure 2a–c, respectively, and in each subplot the red dashed line indicates the results of Scheme 1 and the solid blue line indicates the results of Scheme 2. In Table 3, the mean and standard deviation of the alignment errors have been listed every 100 s during the whole alignment time.

The curves in Figure 2a,b indicate that the two schemes have similar horizontal alignment errors and the same convergence rate, and reached a steady value in the former 100 s. From the statistical results in Table 3, the pitch error was under 0.03° and the roll error was within −0.03° in the two schemes. Additionally, the standard deviation was around 0.002°, which indicates that the stability of the horizontal alignment results of the two methods was equivalent. However, the yaw error in Figure 2c shows that the noise property of Scheme 1 was obvious, while the sound characteristic of Scheme 2 was stable. The standard deviation of yaw errors of Scheme 2 shows that the value was around 0.005° after 300 s. In the contrast, the standard deviation of the yaw of Scheme 1 in Table 3 did not reach the stable value during the whole alignment. That is, the convergence property of Scheme 2 was better than that of Scheme 1.

Notice that in Table 3, although the stability of Scheme 2 was improved, the constant bias of the accelerometer was accumulating during the integral operation. It can be seen that this error contaminates the final results of the self-alignment, and it affects the directional deviation of Scheme 2. The defects of the above two schemes reduce the performance of general quaternion self-alignment. According to the aforementioned analysis, the two schemes were all suboptimal. To improve the performance of general quaternion self-alignment, a novel method based on Kalman filtering technology is proposed in the next section.

## 3. Self-Alignment Based on a Kalman Filter

In this section, a Kalman filter based on minimum variance theory is designed for quaternion self-alignment, and the quaternion pseudo-measurement model is also investigated. By the adaptive filtering technology, the state-dependent noises in observation vector are attenuated. Since $q_{b0}^{n0}$ is a constant quantity, the process equation of the filter is noise-free, and the algorithm is developed as follows.

### 3.1. The Quaternion Pseudo-Measurement Model

It is assumed that the pair of 3 × 1 unit column-vector $\beta_{k}$ and $\alpha_{k}$ are obtained at the time instant k, and they are related by the quaternion of rotation $q_{n0}^{b0}$ as follows:

(15) $$\beta_{k}=q_{n0}^{b0}⊗\alpha_{k}⊗{\left(q_{n0}^{b0}\right)}^{*}$$

Post-multiplying Equation (15) by $q_{n0}^{b0}$ leads to the following equation:

(16) $$0=\beta_{k}⊗q_{n0}^{b0}-q_{n0}^{b0}⊗\alpha_{k}$$

where [·⊗] and [·⊙ ] are used to denote the linear mappings from ${\mathbb{R}}^{3\times 1}$ to ${\mathbb{R}}^{4\times 4}$:

(17) $$0=\left(\left[\beta_{k}⊗\right]-\left[\alpha_{k}⊙\;\right]\right)q_{n0}^{b0}$$

where:

(18) $$\{\begin{array}{c} \left[\beta_{k}⊗\right]=\left[\begin{array}{cc} 0 & -\beta_{k}^{T}\\ \beta_{k} & \left[\beta_{k}\times \right] \end{array}\right]\\ \left[\alpha_{k}⊙\;\right]=\left[\begin{array}{cc} 0 & -\alpha_{k}^{T}\\ \alpha_{k} & -\left[\alpha_{k}\times \right] \end{array}\right] \end{array}$$

This equation, which is linear with respect to the quaternion $q_{n0}^{b0}$, is the model equation of an error-free quaternion measurement [22].

In the real system, the observation vectors are the outputs of the inertial sensors which contain unknown noises. In this work, the measurement models of the accelerometer and gyroscope are defined by:

(19a) $${\tilde{f}}^{b}=f^{b}+\nabla^{b}+\epsilon^{b}$$

(19b) $${\tilde{\omega }}^{b}=\omega^{b}+\varepsilon^{b}+\eta^{b}$$

where ${\tilde{f}}^{b}\in {\mathbb{R}}^{3\times 1}$ is the actual output of the accelerometer; $\nabla^{b}\in {\mathbb{R}}^{3\times 1}$ and $\epsilon^{b}\in {\mathbb{R}}^{3\times 1}$, respectively, denote the constant bias and random noise; ${\tilde{\omega }}^{b}\in {\mathbb{R}}^{3\times 1}$ is the actual value of the angular velocity of the *b*-frame with respect to the *i*-frame; $\varepsilon^{b}\in {\mathbb{R}}^{3\times 1}$ and $\eta^{b}\in {\mathbb{R}}^{3\times 1}$ denote the constant bias and random noises in the IMU axes.

Substituting Equations (19a) and (19b) into Equation (7), we rewrite the normalized equation as:

(20) $${\tilde{\beta }}_{k}=C\left(q_{n0}^{b0}\right)\alpha_{k}+\delta \beta_{k}$$

where $\delta \beta_{k}\in {\mathbb{R}}^{3\times 1}$ is the normalized noise.

Due to:

(21) $$\beta_{k}={\tilde{\beta }}_{k}-\delta \beta_{k}$$

From Equations (16) and (21), a modified Equation (17) is given by:

(22) $$0=\left(\left[{\tilde{\beta }}_{k}⊗\right]-\left[\alpha_{k}⊙\;\right]\right)q_{n0}^{b0}-\left[\delta \beta_{k}⊗\right]q_{n0}^{b0}$$

Equation (22) describes a quaternion pseudo-measurement model at time instant k. Unlike the general attitude determination model [22], it is a linear function of the attitude quaternion, and the noise term in Equation (22) is an additive quaternion-dependent vector.

### 3.2. Kalman Filter

It is well known that the linear Kalman filter is an optimal globally-convergent state estimator for the linear state-space model with white noises. However, it becomes suboptimal if the statistics of the measurement noises are unknown, and this is the case of the aforementioned pseudo-measurement model, where the noise vector is quaternion-dependent. The adequate approach for on-line enhancement of the Kalman filter performance is adaptive filtering. The approach presented herein is inspired by [23,24], which is an adaptive-like Kalman filter, and it can cover the state-space model with the correlated noises by an adaptive filter. Using the quaternion pseudo-measurement model, the filtering model for quaternion self-alignment based on the Kalman filter is given by:

(23) $$\{\begin{array}{c} {q_{n0}^{b0}}_{k}={q_{n0}^{b0}}_{k-1}\\ 0=H_{k}{q_{n0}^{b0}}_{k}+\upsilon_{k} \end{array}$$

where ${q_{n0}^{b0}}_{k}\in {\mathbb{R}}^{4\times 1}$ denotes the estimated initial quaternion at time instant k; $H_{k}=\left[{\tilde{\beta }}_{k}⊗\right]-\left[\alpha_{k}⊙\;\right]$ denote the sensitive matrix of the measurement model; $\upsilon_{k}=-\left[\delta \beta_{k}⊗\right]{q_{n0}^{b0}}_{k}$ is the quaternion-dependent noise.

The Kalman filter is summarized as follows:

(24) $$e_{k+1}=-H_{k}{{\hat{q}}_{n0}^{b0}}_{k}$$

(25) $$\Lambda_{k+1}=\Lambda_{k}+\frac{1}{k+1}\left[e_{k+1}e_{k+1}^{T}-\Lambda_{k}\right]$$

(26) $$G_{k+1}=P_{k}H_{k}^{T}\left[H_{k}P_{k}H_{k}^{T}+\Lambda_{k+1}\right]{\;}^{-1}$$

(27) $${{\hat{q}}_{n0}^{b0}}_{k+1}={{\hat{q}}_{n0}^{b0}}_{k}+G_{k+1}e_{k+1}$$

(28) $$P_{k+1}=P_{k}-G_{k+1}\left[H_{k}P_{k}H_{k}^{T}+\Lambda_{k+1}\right]G_{k+1}^{T}$$

where $\Lambda_{k+1}\in {\mathbb{R}}^{4\times 4}$ is an estimate of a covariance of the filtering measurement noise based on k+1 data pairs, and the term $e_{k+1}$ is the object function for the measurement residual process. Due to the pseudo-measurement model, the ideal measurement is vector 0; thus, $e_{k+1}$ is the opposite vector of a priori state estimation. According to the Kalman filtering process, we can find that the filtering process destroyed the normalization of the estimated quaternion, which lowered the convergence rate of the Kalman filter. To overcome the problem, we adopt the brute-force normalization method to keep the validity of the estimated quaternion in this paper [27].

### 3.3. Simulation Test

In this section, a simulation test for the novel quaternion self-alignment method is designed, and it is defined as Scheme 3. For comparative purposes, the simulation test is conducted with the same conditions described in Section 2.4.

Without a loss of generality, the initial attitude quaternion for the Kalman filter was ${{\hat{q}}_{b0}^{n0}}_{0}={\left[0.6690\;0.1853\;0.5090\;0.5090\right]}^{T}$, and the corresponding error attitude at start-up was $\phi ={\left[50{}^{\circ}\;50{}^{\circ}\;50{}^{\circ}\right]}^{T}$, the adaptive measurement noise was $\Lambda_{0}=diag\left[0.1\;0.1\;0.1\;0.1\right]$, and the initial estimation error covariance matrix was $P_{0}=diag\left[10000\;10000\;10000\;10000\right]$.

**Figure 3:**
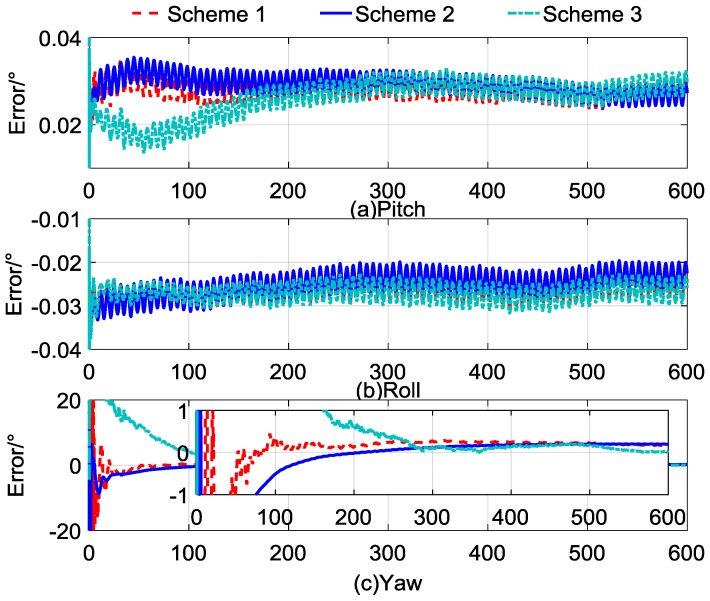
Curves of self-alignment errors.

The simulation time was 600 s, and the results compared with the Schemes 1 and 2 are shown in Figure 3a–c, indicating the errors of pitch, roll, and yaw, respectively. To show the results clearly, we use the red dashed line to represent the results of Scheme 1, the blue line to represent the results of Scheme 2, and the cyan dashed, dotted line to represent the results of Scheme 3. In the interest of brevity, the statistics for the alignment errors of Schemes 2 and 3 are listed in Table 4, and the statistical results of Scheme 1 are consistent with Table 3.

In Figure 3a,b, we can find that the horizontal alignment results of Scheme 3 show a similar accuracy after 300 s compared with the other two methods, and there are no accumulated errors in Scheme 3. According to the partial enlarged views of Figure 3c, the accuracy of Scheme 3 has an advantage over the other two methods in the final results of self-alignment, which is also proved by the statistics for the alignment errors in Table 4. As can be seen in Table 4, when the alignment time lasted for 600 s, the alignment error of the yaw of Scheme 3 was 0.0658°, while the alignment errors of yaw of Schemes 1 and 2 were 0.1911° and 0.2002°. It can be found that the alignment errors of yaw of Scheme 3 were lower than those of Schemes 1 and 2, and the adaptive filter was optimal for the state-dependent measured model.

With comparing the results of the Scheme 3 to the other two methods, although the accuracy of the novel quaternion self-alignment method was improved, the convergence rate was poor because of the random noise which was incorporated into the observation vector, and the stability of the proposed method was also poor. In addition, the statistics for the yaw error in Table 4 indicated that the standard deviation of Scheme 3 was greater than that of Scheme 2. These defects weaken the practical performance of the new method. To address these shortcomings, the parameter recognition and vector reconstruction algorithm are investigated for novel quaternion self-alignment, and the method is illustrated in detail in the next section.

## 4. Improvement to the Observation Vectors

In this section, we drive the reconstructed observation vectors to improve the convergence rate of the estimation of the novel quaternion self-alignment method based on a Kalman filter. Making use of the slow-varying characteristic of the observation vectors, the random noise of the observation vector is restrained, and the reconstructed vectors contain the gravitational apparent motion information. This superior characteristic is contributory for extracting the effective information from the observation vectors, and it makes the algorithm practical.

### 4.1. The Model of Parameter Recognition

According to Equations (6) and (7), the theoretical expression of the gravitational apparent motion on the swaying base is given by:

(29) $$o=q_{e0}^{b0}⊗q_{e}^{e0}⊗q_{n}^{e}⊗g^{n}⊗{\left(q_{n}^{e}\right)}^{*}⊗{\left(q_{e}^{e0}\right)}^{*}⊗{\left(q_{e0}^{b0}\right)}^{*}$$

where the quaternion of rotation $q_{e0}^{b0}$ indicates the orientation of the *b*0-frame relative to the *e*0-frame, $q_{e}^{e0}$ is the attitude quaternion from the *e*-frame to the *e*0-frame, $q_{n}^{e}$ represents the attitude quaternion due to the rotation of the *e*-frame relative to the *n*-frame, $g^{n}={\left[\begin{array}{ccc} 0 & 0 & g \end{array}\right]}^{T}$.

In Equation (29), it can be found that only $q_{e}^{e0}$ is the time-varying quaternion, and thus Equation (29) can be rewritten as:

(30) $$o=\left[\begin{array}{ccc} a_{11} & a_{12} & a_{13}\\ a_{21} & a_{22} & a_{23}\\ a_{21} & a_{32} & a_{33} \end{array}\right]\left[\begin{array}{ccc} \cos\left(\omega_{ie}t\right) & -\sin\left(\omega_{ie}t\right) & 0\\ \sin\left(\omega_{ie}t\right) & \cos\left(\omega_{ie}t\right) & 0\\ 0 & 0 & 1 \end{array}\right]\left[\begin{array}{ccc} b_{11} & b_{12} & b_{13}\\ b_{21} & b_{22} & b_{23}\\ b_{31} & b_{32} & b_{33} \end{array}\right]\left[\begin{array}{c} 0\\ 0\\ g \end{array}\right]$$

where $a_{ij}$ and $b_{ij}\left(i=1,2,3;j=1,2,3\right)$ indicate the constant values, and they are the elements of the direction cosin matrices which consists of $q_{e0}^{b0}$ and $q_{n}^{e}$. It can be found that the theoretical observation vector is relative to the rotation of the Earth, and it is a slow-varying vector. 

With the matrix operation, the simplified form of Equation (29) can be given by:

(31) $$o=\left[\begin{array}{ccc} \gamma_{11} & \gamma_{12} & \gamma_{13}\\ \gamma_{21} & \gamma_{22} & \gamma_{23}\\ \gamma_{31} & \gamma_{32} & \gamma_{33} \end{array}\right]\left[\begin{array}{c} \cos\left(\omega_{ie}t\right)\\ \sin\left(\omega_{ie}t\right)\\ 1 \end{array}\right]$$

where $\tilde{o}$ indicates the actual value of the observation, and δo denotes the measurement noise.

According to the above Equation (31), the noise-free ideal observation vector can be obtained. However, as the result of the constant parameter $\gamma_{ij}$ is unknown, it is difficult to calculate the observation vector with Equation (31) directly. Thanks to the parameter recognition technology, the unknown constant parameter $\gamma_{ij}$ can be estimated by the recursive least squares algorithm. In this work, the parameter recognition model of the observation vectors is given by:

(32) $$\{\begin{array}{c} \gamma_{k+1}=\gamma_{k}\\ {\tilde{o}}_{k+1}=\gamma_{k+1}M_{k+1}+\delta o_{k+1} \end{array}$$

where $M_{k+1}={\left[\cos\left(\omega_{ie}t_{k+1}\right)\;\sin\left(\omega_{ie}t_{k+1}\right)\;1\right]}^{T}$. Due to the slow rotation rate of the Earth and the shorter duration process of the self-alignment, the observation vectors are the slow-varying parameters. Therefore, we can remove the random noise by the recursive least squares technology, which is computationally efficient. The detailed statement of this method is analyzed in the next subsection.

### 4.2. The Observation Vector Reconstructed Algorithm

According to the aforementioned parameter recognition model, the on-line estimation algorithm based on the recursive least squares algorithm (RLS) is adopted to avoid large data storage and heavy computation. Due to the independent properties of the three elements of the observation vector, we can take the recognition for the *z*-axis as an example, and the parameter recognition model Equation (32) can be rewritten as:

(33) $$\{\begin{array}{c} \gamma_{z,k+1}=\gamma_{z,k}\\ {\tilde{o}}_{z,k+1}=M_{k+1}^{T}\gamma_{z,k+1}+\delta o_{z,k+1} \end{array}$$

where $\gamma_{z,k+1}=\left[\begin{array}{ccc} \gamma_{31} & \gamma_{32} & \gamma_{33} \end{array}\right]$, ${\tilde{o}}_{z,k+1}$ indicates the *z*-axis element of the measured observation vectors.

With the previous development, the RLS algorithm for parameter recognition is summarized as follows:

(34) $$\{\begin{array}{c} K_{z,k+1}=P_{z,k}M_{z,k+1}{\left(M_{k+1}^{T}P_{z,k}M_{k+1}+R_{z,k+1}\right)}^{-1}\\ {\hat{\gamma }}_{z,k+1}={\hat{\gamma }}_{z,k}+K_{z,k+1}\left({\tilde{o}}_{z,k+1}-M_{k+1}^{T}{\hat{\gamma }}_{z,k}\right)\\ P_{z,k+1}=\left(I_{k+1}-K_{z,k+1}M_{k+1}^{T}\right)P_{z,k} \end{array}$$

Based on the aforementioned analysis, the optimal constant parameters ${\hat{\gamma }}_{z,k+1}$ can be estimated. According to the similar approach, we can obtain the other estimated parameters ${\hat{\gamma }}_{x,k+1}$ and ${\hat{\gamma }}_{x,k+1}$. Making use of the estimated parameters ${\hat{\gamma }}_{k+1}$, the new observation vectors can be reconstructed by:

(35) $${\hat{o}}_{k+1}={\hat{\gamma }}_{k+1}M_{k+1}$$

For more clarity, the proposed self-alignment algorithm using the reconstructed observation vectors is listed in Table 5.

### 4.3. Simulation Test

In this subsection, the simulation test is described for self-alignment based on a Kalman filter, where the improved measurements are used to construct the observation vectors, and the new method is defined as Scheme 4. For the purpose of comparison, swinging parameters and sensor errors are shown in Table 1 and Table 2, respectively. The sampling rate of the outputs of the inertial sensors was 100 Hz. In addition, the filtering initialization of the Kalman filter was set to the same parameters shown in Section 3.3. The initial parameters for RLS were defined as: ${\hat{\gamma }}_{i,0}=0_{3}$, $P_{i,0}=diag\left[\begin{array}{ccc} 10000 & 10000 & 10000 \end{array}\right]$, $R_{i,0}=500\mathrm{ug}$, where i=x,y,z.

**Figure 4:**
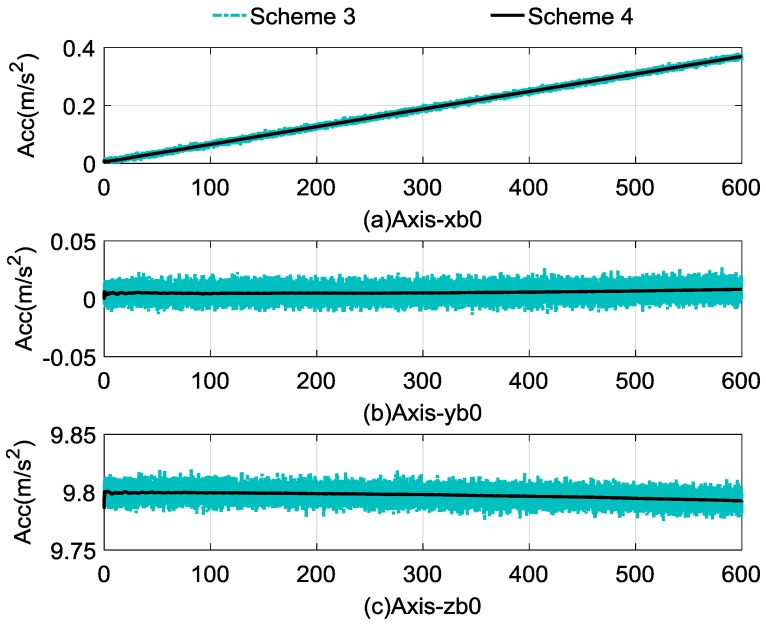
Comparison between calculated and reconstructed gravitational apparent motion.

**Figure 5:**
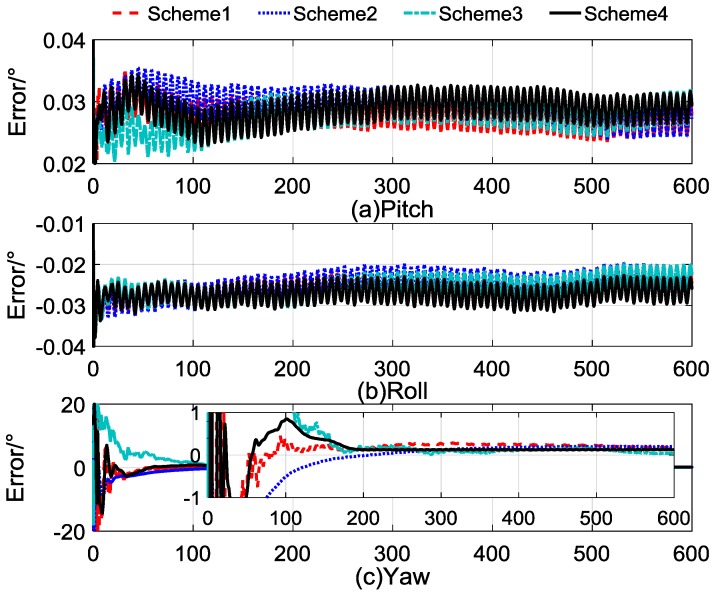
Curves of alignment errors.

The self-alignment process lasted for 600 s. The comparison between the calculated and reconstructed gravitational apparent motion is shown in Figure 4, and the alignment errors compared with Schemes 1–3 are shown in Figure 5. In Figure 4, the cyan dashed dotted line denotes the noised observation vector and the black curves denote reconstructed vectors, and Figure 4a–c denote the *x*-axis, *y*-axis, and *z*-axis, respectively. Figure 5a–c denote the alignment errors of the pitch, roll, and yaw. In order to show this clearly, the red dashed line represents the results of Scheme 1, the blue dotted line represents the results of Scheme 2, the cyan dashed, dotted line represents the results of Scheme 3, and the black line represents the results of Scheme 4. Table 4 lists the statistical results of Schemes 3 and 4; the statistical results of Schemes 1 and 2 are equivalent with those shown in Table 1, which can be used as the comparable results.

Figure 4 reveals that the observation vectors are slowly varying, and the varying trends are consistent with the rotation rate of the Earth. It shows that the random noises of the observation vectors of Scheme 4 have been eliminated effectively, and the useful information is retained, which contributes to the fast convergence rate.

The horizontal alignment errors, which are shown in Figure 5a,b revealed that the accuracy and convergence rate of the four schemes are equivalent. The main priority of Scheme 4 is the performance of the yaw alignment results. In Figure 5c, we can see that Scheme 4 has a faster convergence rate than the other three schemes, and it possesses more stable characteristics after 200 s.

Table 6 shows the statistical results of Schemes 3 and 4, and the statistical results of Schemes 1 and 2 are equivalent to those shown in Table 3. In Table 6, the statistical results show that Scheme 4 has the same stability as Scheme 2 and the same accuracy as Scheme 3 at the end of the self-alignment. The standard deviation of the yaw error of the Scheme 4 was less than 0.004°, and the error of the yaw was around 0.12° when the self-alignment lasted for 200 s, while the standard deviation of the yaw error of Scheme 3 was larger than 0.01°. Thus, we can conclude that the convergence rate and stability of Scheme 4 are obviously improved. However, it can be found that the error mean of the yaw of Scheme 3 was 0.0706° at the end of the self-alignment; thus, the error caused by Scheme 3 was smaller than that caused by Scheme 4, which was not an expected result. This is because the error of the yaw of Scheme 3 did not converge to a stable value, and the error fluctuated, so the error may be smaller at an interval.

The simulation test showed the superior performance of Scheme 4, but the simulated data was generated in an ideal situation, and the errors of the inertial sensors were assumed as white noise, which is not consistent with the real system. In order to verify the practical application and the performance of the adaptive filter for the complicated noises of the inertial sensors, the turntable test is undertaken in the next section.

## 5. Turntable Test

**Figure 6:**
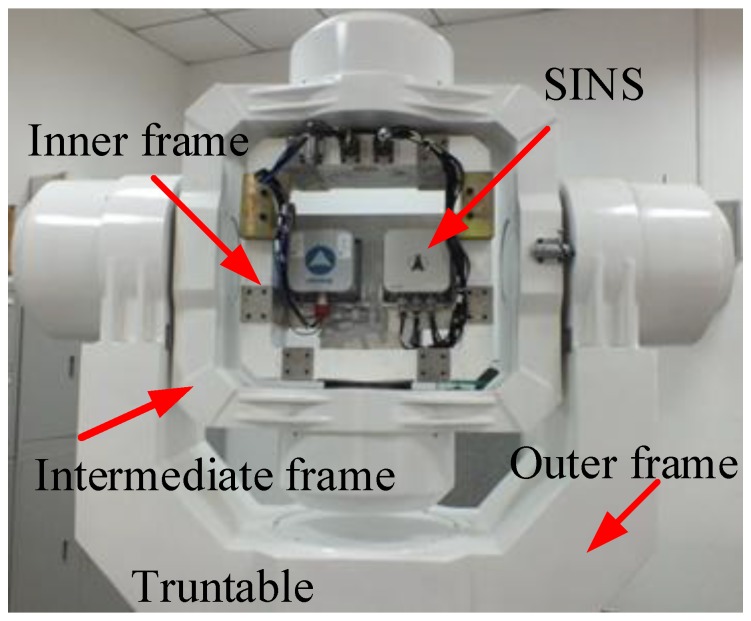
Turntable and SINS.

For the turntable test, the equipment was installed as shown in Figure 6; the rate controlling accuracy of the turntable of this work was ±0.0005°/s, and the angle controlling accuracy was ±0.0001°. Additionally, the angle information could be provided via the serial communication port as a response to the external time-synchronization signal. In this test, the inner, intermediate, and outer frames were used to simulate the vehicle’s roll, pitch, and yaw, respectively. The SINS used in this test is a navigation-grade SINS, in which there are flexible gyros and quartz accelerometers, and the precision of the inertial sensors is listed in Table 7. According to [28], the sensor’s coupling coincident scale factors, installing error, system error, and the variables of the fiber-optic gyro related to the gravity can be calculated exactly and compensated by a calibration test. Thus, if the test is executed after the calibration test with the same startup of the SINS, the above-mentioned existence errors of SINS can be ignored.

**Figure 7:**
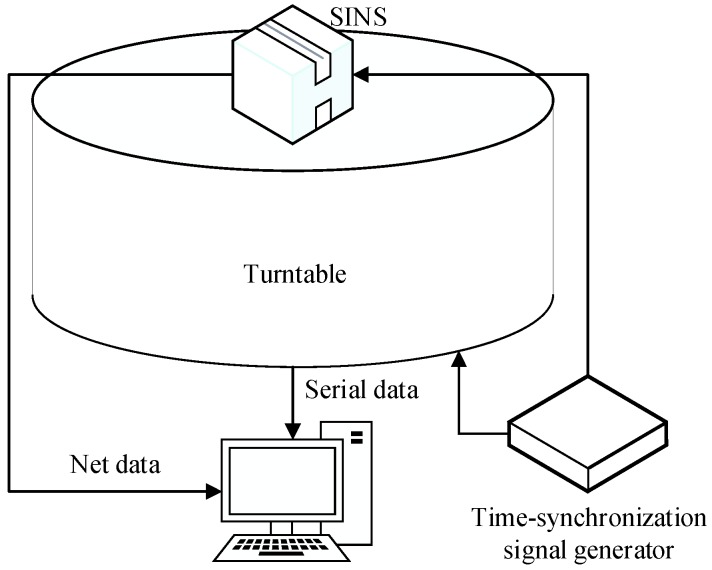
Construction of the turntable test.

The construction of the turntable test is shown in Figure 7. As can be seen in Figure 7, when the time-synchronization signal was generated, the current attitude angle data of the turntable was sent to the navigation computer via the serial port. Meanwhile, the current IMU outputs were collected, and the alignment results were acquired by the embedded algorithm. All effective data was stored by the navigation computer at 200 Hz, which is the frequency of the trigger signal for the turntable and the update frequency of the IMU outputs. Meanwhile, the alignment solution was carried out and saved at every sampling interval.

**Figure 8:**
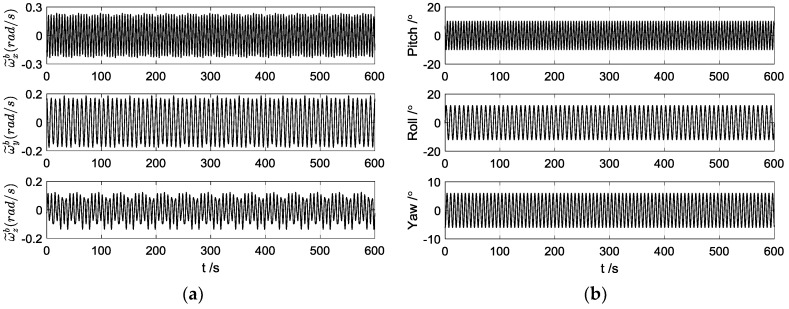
(**a**) Curves of the measured angular rates; (**b**) Curves of the actual attitude angles.

**Figure 9:**
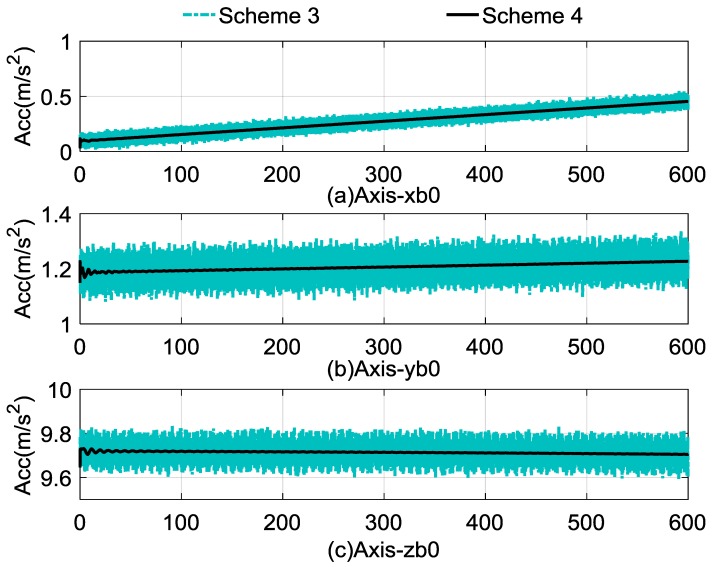
Comparison between calculated and reconstructed gravitational apparent motion.

**Figure 10:**
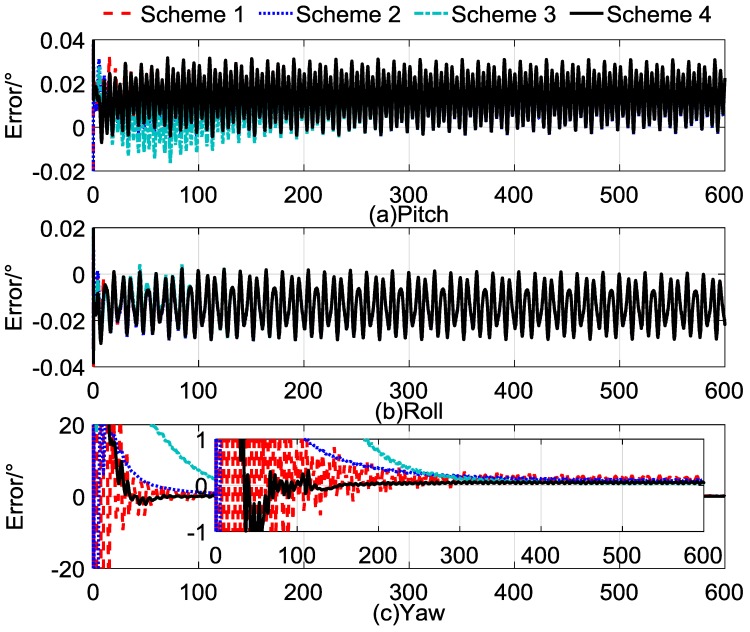
Curves of alignment errors.

The swaying parameters of the turntable were still set as in Table 1, and the angular rates and the actual attitude angles are shown in Figure 8. The calculated and reconstructed gravitational apparent motion of the turntable tests are shown in Figure 9, and the alignment errors are depicted in Figure 10.

In Figure 9, the cyan dashed dotted line represents the observation vectors of Scheme 3 and the black line represents the reconstructed observation vectors, and Figure 9a–c denote the apparent gravitation of the *x*-axis, *y*-axis, and *z*-axis, respectively. It is obvious that the computed measured observation vector was slowly changing along with the alignment process, and this is coincident with the above analysis. Due to this characteristic of the observation vector, the reconstructed observation vector was estimated by the RLS method. It can be found that the random noises in the observation vector were eliminated effectively, which will be helpful to speed up the alignment process.

The results of the turntable tests are shown in Figure 10a–c, indicating the errors of the pitch, roll, and yaw, respectively. Due to the swaying motion of the turntable, the alignment results were oscillating with small amplitude. In Figure 10a,b, the error of the pitch and the roll of the four schemes converge rapidly, which is coincident with the results of the simulation test in Section 4.3. The error of the yaw in Figure 10c shows that Scheme 4 had a better performance than the other three schemes in terms of the convergence rate and alignment accuracy, and the stability of the alignment results of Scheme 4 was better than that of the others.

Table 8 shows the statistics for the alignment errors of Schemes 3 and 4. It is shown that the errors of the pitch and roll of Schemes 3 and 4 were reduced to less than 0.015° in 100 s, and the standard deviation of the level angles was below 0.01° in 100 s. The error of the yaw of Scheme 3 was reduced to less than 0.1° in 400 s, and the standard deviation was reduced to less than 0.02° in 400 s. In comparison, the yaw error of Scheme 4 was reduced to less than 0.1° in 200 s and the standard deviation was reduced to less than 0.02° in 200 s. These features validate the correctness of the aforementioned analysis and implemented algorithms, and they showed the sound performance of Scheme 4 in practice.

## 6. Conclusions

This paper studied the general quaternion self-alignment method based on the ***q***-method principle, and an improved algorithm based on the velocity apparent motion was designed. However, the analysis and simulation indicated that: (1) in the general quaternion self-alignment method, the alignment accuracy and convergence rate are easily affected by the random noise of the inertial sensors; and (2) although the random noises are eliminated effectively by the improved algorithm, using the apparent velocity motion, the alignment result drifts because of the cumulative effect of the constant drift of the inertial sensors.

Based on general quaternion self-alignment, an improved method adopting the optimal estimation theory was investigated, in which a quaternion pseudo-measurement model with the state-dependent noises was established. A Kalman filter with adaptive filter characteristics was studied, and parameter recognition and observation vector reconstruction technology were adopted in the proposed method. Simulations and turntable tests indicated that the alignment accuracy and convergence rate of the yaw were improved. The algorithms proposed in this paper could be useful in many applications which require aligning SINS on the swaying base, such as when mooring ships. In future works, we will further test the algorithms on moving vehicles and try to handle them with large-motion maneuvers.

## Figures and Tables

**Table 1:** Swinging parameters.

	Pitch	Roll	Yaw
Amplitude (°)	10	12	6
Frequency (Hz)	0.2	0.125	0.15
Initial phase (°)	0	0	0
Swaying center (°)	0	0	0

**Table 2:** Sensor errors.

	Gyro Noise (°/h)		Accelerometer Noise (µg)	
	Constant	Random	Constant	Random
*x*-axis	0.05	0.05	500	500
*y*-axis	0.05	0.05	500	500
*z*-axis	0.05	0.05	500	500

**Table 3:** Statistics for alignment errors (°).

Time(s)			1–100	101–200	201–300	301–400	401–500	501–600
Scheme 1	Pitch	Mean	0.0287	0.0286	0.0279	0.0275	0.0272	0.0279
		Std	0.0023	0.0020	0.0020	0.0020	0.0019	0.0019
	Roll	Mean	−0.0284	−0.0274	−0.0272	−0.0266	−0.0259	−0.0250
		Std	0.0025	0.0022	0.0021	0.0021	0.0021	0.0022
	Yaw	Mean	0.1254	0.3161	0.2955	0.2404	0.2044	0.1918
		Std	6.7385	0.0411	0.0252	0.0137	0.0106	0.0049
Scheme 2	Pitch	Mean	0.0301	0.0296	0.0283	0.0272	0.0264	0.0271
		Std	0.0024	0.0020	0.0020	0.0020	0.0019	0.0019
	Roll	Mean	−0.0281	−0.0271	−0.0265	−0.0256	−0.0246	−0.0233
		Std	0.0028	0.0022	0.0021	0.0021	0.0021	0.0022
	Yaw	Mean	−1.6399	0.1368	0.2546	0.2611	0.2469	0.2307
		Std	4.1810	0.0752	0.0114	0.0050	0.0059	0.0051

**Table 4:** Statistics for alignment errors (°).

Time(s)			1–100	101–200	201–300	301–400	401–500	501–600
Scheme 2	Pitch	Mean	0.0309	0.0299	0.0300	0.0295	0.0281	0.0268
		Std	0.0028	0.0019	0.0018	0.0018	0.0019	0.0017
	Roll	Mean	−0.0284	−0.0265	−0.0242	−0.0239	−0.0248	−0.0230
		Std	0.0026	0.0024	0.0024	0.0022	0.0022	0.0021
	Yaw	Mean	−2.8787	−0.1752	0.0612	0.1564	0.1917	0.2002
		Std	7.8888	0.1344	0.0403	0.0166	0.0069	0.0035
Scheme 3	Pitch	Mean	0.0255	0. 0239	0.0280	0.0293	0.0279	0.0285
		Std	0.6458	0.0031	0.0022	0.0020	0.0021	0.0021
	Roll	Mean	−0.0255	−0.0275	−0.0265	−0.0271	−0.0279	−0.0263
		Std	0.1561	0.0019	0.0021	0.0022	0.0022	0.0022
	Yaw	Mean	13.7246	1.6706	0.3349	0.0955	0.1714	0.0658
		Std	8.3981	0.9930	0.1704	0.0335	0.0201	0.0496

**Table 5:** Self-alignment algorithm based on reconstructed observation vectors.

**Initialization**	k = 1, $q_{b}^{b0}=q_{n}^{n0}=\left[1,0,0,0\right]$.
Step 1:	k = k + 1;
Step 2:	Update $q_{n}^{n0}\left(t\right)$ and $q_{b}^{b0}\left(t\right)$ by Equation (2);
Step 3:	Compute ${\tilde{o}}_{k}$ and by r Equation (7);
Step 4:	Compute ${\hat{o}}_{k}$ by Equations (33)–(35);
Step 5:	Compute ${\tilde{\beta }}_{k}$ and $\alpha_{k}$ by normalizing ${\hat{o}}_{k}$ and r;
Step 6:	Compute ${{\hat{q}}_{n0}^{b0}}_{k}$ by Kalman Filter (see Equations (23)–(28));
Step 7:	Obtain the attitude matrix at current time (see (1));
Step 8:	Go to Step 1 until the end.

**Table 6:** Statistics for alignment errors (°).

Time(s)			1–100	101–200	201–300	301–400	401–500	501–600
Scheme 3	Pitch	Mean	0.0274	0.0272	0.0289	0.0293	0.0282	0.0284
		Std	0.2474	0.0024	0.0020	0.0020	0.0020	0.0021
	Roll	Mean	−0.0255	−0.0275	−0.0265	−0.0271	−0.0279	−0.0263
		Std	0.1561	0.0019	0.0021	0.0022	0.0022	0.0022
	Yaw	Mean	5.6506	0.4862	0.1323	0.0835	0.1377	0.0706
		Std	4.8208	0.3485	0.0534	0.0233	0.0141	0.0321
Scheme 4	Pitch	Mean	0.0313	0. 0270	0. 0291	0.0299	0.0295	0.0288
		Std	0.2474	0.0022	0.0020	0.0018	0.0019	0.0017
	Roll	Mean	−0.0265	−0.0276	−0.0266	−0.0271	−0.0279	−0.0263
		Std	0.1350	0.0021	0.0021	0.0022	0.0022	0.0022
	Yaw	Mean	−0.3631	0.3812	0.1238	0.1249	0.1252	0.1263
		Std	4.0402	0.2066	0.0039	0.0037	0.0038	0.0035

**Table 7:** Sensor parameters.

Gyroscope			
Constant bias	<0.01°/h(1σ)	Nonlinearity of scale factor	≤50ppm(1σ)
Repetitiveness of constant bias	<0.01°/h(1σ)	Repetitiveness of scale factor	≤50ppm(1σ)
Random walk	$<0.005{}^{\circ}/\sqrt{h}$	Measuring range	−300~+300^°^/s
**Accelerometer**			
Measuring range	−20~+20 g	Bias	<5 × 10^−4^ g
Threshold	<5 × 10^−6^ g	Temperature coefficient of bias	<6 × 10^−5^/^°^C
			(−40~+40 ^°^C)
Repetitiveness of scale factor	$<3.5\times 10^{-5}g\left(1\sigma \right)$	Repetitiveness of bias	$<2.5\times 10^{-4}g\left(1\sigma \right)$
Temperature coefficient of Scale factor	<6 × 10 ^−5^/^°^C	Bandwidth	>800 Hz
	(−40~+40 ^°^C)		

**Table 8:** Statistics for alignment errors (°).

Time(s)			1–100	101–200	201–300	301–400	401–500	501–600
Scheme 3	Pitch	Mean	0.0049	0.0096	0.0129	0.0137	0.0139	0.0141
		Std	0.1363	0.0097	0.0095	0.0093	0.0092	0.0089
	Roll	Mean	−0.0119	−0.0135	−0.0138	−0.0138	−0.0139	−0.0139
		Std	0.0429	0.0080	0.0081	0.0078	0.0079	0.0075
	Yaw	Mean	21.373	2.6712	0.3510	0.1004	0.0556	0.0496
		Std	16.5760	1.7737	0.1599	0.0281	0.0133	0.0120
Scheme 4	Pitch	Mean	0.0139	0.0139	0.0138	0.0140	0.0141	0.0144
		Std	0.1362	0.0096	0.0095	0.0093	0.0092	0.0089
	Roll	Mean	−0.0128	−0.0134	−0.0136	−0.0137	−0.0139	−0.0140
		Std	0.0429	0.0081	0.0081	0.0078	0.0079	0.0075
	Yaw	Mean	5.9610	0.0040	0.0466	0.0586	0.0585	0.0588
		Std	11.4950	0.0748	0.0139	0.0124	0.0122	0.0124

## Appendix A

The coordinate frames used in this paper are defined as follows:

1. *n*-frame: Orthogonal reference frame aligned with east–north–up(ENU) geodetic axes;
2. *n*0-frame: Orthogonal reference frame non-rotating relative to the *i*-frame, which is formed by fixing the frame n at start-up in the inertial space;
3. *b*-frame: Orthogonal reference frame aligned with inertial measurement unit (IMU) axes;
4. *b*0-frame: Orthogonal reference frame non-rotating relative to the *i*-frame, which is formed by fixing the frame b at start-up in the inertial space;
5. *e*-frame: Earth-centered Earth-fixed (ECEF) orthogonal reference frame;
6. *e*0-frame: Orthogonal reference frame non-rotating relative to the *i*-frame, which is formed by fixing the frame e at start-up in the inertial space; and
7. *i*-frame: Earth-centered initially-fixed orthogonal reference frame.
